# Molecular Insights into the Interaction of RONS and Thieno[3,2-c]pyran Analogs with SIRT6/COX-2: A Molecular Dynamics Study

**DOI:** 10.1038/s41598-018-22972-9

**Published:** 2018-03-19

**Authors:** Dharmendra K. Yadav, Surendra Kumar, Saloni, Sanjeev Misra, Lalit Yadav, Mahesh Teli, Praveen Sharma, Sandeep Chaudhary, Naresh Kumar, Eun Ha Choi, Hyung Sik Kim, Mi-hyun Kim

**Affiliations:** 1 College of Pharmacy, Gachon University of Medicine and Science, 191, Hambangmoe-ro, Yeonsu-gu, Incheon, 21936 Republic of Korea; 2 Department of Biochemistry, All India Institute of Medical Science, Jodhpur, Rajasthan 342005 India; 3 Department of Chemistry, Malaviya National Institute of Technology, Jawaharlal Nehru Marg, Jaipur, 302017 India; 4 Faculty of Biochemistry and Molecular Medicine Aapistie, University of Oulu, 7A, Oulu, 90220 Finland; 5 Plasma Bioscience Research Center/Department of Electrical and Biological Physics, Kwangwoon University, 20 Kwangwon-Ro, Nowon-Gu, Seoul, 139-701 Republic of Korea; 6 School of Pharmacy, Sungkyunkwan University, Suwon, 16419 Republic of Korea; 7 Present Address: Department of Chemistry, Research group PLASMANT, University of Antwerp, BE-2610 Wilrijk-Antwerp, Belgium

## Abstract

SIRT6 and COX-2 are oncogenes target that promote the expression of proinflammatory and pro-survival proteins through a signaling pathway, which leads to increased survival and proliferation of tumor cells. However, COX-2 also suppresses skin tumorigenesis and their relationship with SIRT6, making it an interesting target for the discovery of drugs with anti-inflammatory and anti-cancer properties. Herein, we studied the interaction of thieno[3,2-c]pyran analogs and RONS species with SIRT6 and COX-2 through the use of molecular docking and molecular dynamic simulations. Molecular docking studies revealed the importance of hydrophobic and hydrophilic amino acid residues for the stability. The molecular dynamics study examined conformational changes in the enzymes caused by the binding of the substrates and how those changes affected the stability of the protein-drug complex. The average RMSD values of the backbone atoms in compounds **6** and **10** were calculated from 1000 ps to 10000 ps and were found to be 0.13 nm for both compounds. Similarly, the radius of gyration values for compounds **6** and **10** were found to be 1.87 ± 0.03 nm and 1.86 ± 0.02 nm, respectively. The work presented here, will be of great help in lead identification and optimization for early drug discovery.

## Introduction

Skin cancer is the most common cancer in the United States^1,2^. The development of skin cancer requires both genomic and proteomic alterations. Specifically, significant oncogenic or tumor-suppressive gene modulation may augment cell survival and proliferation, and additionally induce inflammation *in vivo*^3–5^. SIRT6 is a well-known anti-aging protein, which is important for many aspects of cellular homeostasis, including longevity^6–8^. SIRT6 knockout mice have demonstrated genomic instability leading to accelerated premature aging^9^. SIRT6 overexpression in mice has been correlated to an increased lifespan in male mice but not in female mice^10,11^. Furthermore, SIRT6 promotes the reprogramming of induced pluripotent stem cells^12^ and adjusts collagen metabolism in dermal fibroblasts to control skin aging^13^. SIRT6 is predicted to act as a tumor suppressor principally because of its longevity potential and its regulation of genomic integrity. Certainly, it is reported to promote tumorigenesis in the liver and intestinal cancer in a mouse model^14–16^. SIRT6 has also been reported as an oncogene in skin cancer^17^ and prostate cancer^18^, and skin-cancer causing UVB rays reportedly induce SIRT6 expression by augmenting the AKT pathway. Furthermore, SIRT6 enhances the expression of the oncogenic and proinflammatory protein COX-2 by inhibiting AMPK signaling, leading to an increase in cell survival and proliferation, as was demonstrated in a study on the effects of UV radiation^19,20^. COX-2 is an inducible enzyme, which catalyzes the synthesis of prostaglandins, and its up-regulation has been reported in human skin cancer^21^. The genetic ablation of COX-2 was demonstrated to suppress skin tumorigenesis in chemical- and UV-carcinogenesis models^22,23^. Recent studies have revealed that COX-2 activity in intrinsic keratinocytes plays a major role in skin cancer^24^.

The up-regulation of COX-2 in malignant tumors or after acute UV exposure leads to increased PGE2 production followed by the activation of prostaglandin E (EP) receptor signaling, which leads to increased vascular permeability, epidermal proliferation, angiogenesis, and the induction of inflammation. COX enzyme activity is oxidative in nature and can lead to the production of reactive oxygen species (ROS) in activated inflammatory cells, which contributes to a pro-oxidant state^25^. All these lethal effects of chronic COX-2 up-regulation along with UV-induced p53 mutations combine to make a probable cause for the carcinogenic progression instigated and encouraged by chronic UV exposures. UV exposed skin cells generate a number of reactive oxygen and nitrogen species (RONS). However, the effect of generated RONS is regulated by host anti-oxidative defense mechanisms. High levels of H_2_O_2_ or severe oxidative stress were shown to cause increased proteasomal degradation of SIRT1, leading to cellular apoptosis. Similarly, exposure of human monocytes to high doses of H_2_O_2_, or human lung epithelial cells to H_2_O_2_, aldehyde-acrolein, and cigarette smoke extract significantly decreased SIRT1 activity and gene expression. H_2_O_2_ exposure (100 *μ*M, 30 min) caused a drastic drop of 50% in SIRT1 activity, while low doses of H_2_O_2_ had no effect^26^. RONS has also been implicated in the modulation of several proteins linked to the SIRT gene family^27–30^.

The generation of various RONS in a controlled manner under various gases (O_2_, N_2_, He, etc.) is a new approach that is being used in cold-atmospheric thermal plasmas (CAPs). Thus using CAPs might serve as therapeutic areas for treating inflammation-induced cancer. The RONS includes H_2_O_2_, NO_2_, N_2_O, NO_3_, N_2_O_3_, and N_2_O_5_ species and their excessive production aggravates diverse physiological states, including oxidative stress-induced inflammation, which leads to the apoptosis of cancer cells^31,32^. Therefore, therapeutic drugs that inhibit COX-2 and that use CAPs to target SIRT may be an effective hybrid treatment strategy for non-melanoma skin cancers (NMSCs)^33^. Our continuing research is focused on the design and synthesis of a scaffold-based fused heterocyclic such as thieno[3,2-c]pyridin^34^, thia-and oxa-thia[5]helicenes^35^, thieno[3,2-c]pyran^36^, tetra substituted thiophenes^37^, benzo[*h*]quinolines^38^, and a new series of analogs based on thieno[3,2-c]pyran. The present study focuses on two objectives. The first is to explore the structure-activity relationship of SIRT6 with our in-house designed thieno[3,2-c]pyran analogs by considering the active-site ligand binding interaction. The second objective is to study the interaction and reactivity of various RONS with SIRT6 and COX-2 target proteins. Moreover, the relative stability and reactivity of designed thieno[3,2-c]pyran analogs and RONS were studied with the aid of unrestrained molecular dynamic simulation.

## Results and Discussions

### Pharmacokinetic parameters compliance

All of the designed thieno[3,2-c]pyran analogs possessed a good number of hydrogen bond donors and acceptors. Most of the designed compounds possess at least one hydrogen donor and 4 to 7 hydrogen acceptors. The analogs were designed so as to increase the binding affinity of the drug to the receptor mainly by hydrogen bonding^39–44^. The thieno[3,2-c]pyran moiety was added to increase the binding of the designed molecules with the target receptor. The pharmacokinetic parameters suggest that these analogs were found to follow Lipinski’s rule of 5 since it would increase drug-likeness (Table 1). The polar surface area was calculated to optimize the drug’s ability to permeate the cell membrane. Further, lipophilicity (ratio of octanol solubility to water solubility) was calculated as logP, which has been implicated in blood-brain barrier penetration and permeability prediction^45,46^. Metabolism and excretion of xenobiotic compounds from the human body depends on MW and logP^46,47^.

**Table 1:** Compliance of designed thieno[3,2-c]pyran analogues and standard drugs to computational parameters of drug-likeness and ADME properties.

Compounds	Pharmacokinetic property (ADME) dependent on chemical descriptors								Rule of 5 violation
	Oral bioavailability: TPSA (Å^2^)	MW	Log P	Amine group count	H-bond donor		H-bond acceptor		
					Sec-amine group count	Hydroxyl group count	Nitrogen atom count	Oxygen atom count	
01	108.84	359.353	3.15	1	0	0	1	6	0
02	108.84	385.391	3.23	1	0	0	1	6	0
03	103.05	360.338	3.45	0	0	1	0	7	0
04	106.33	326.326	2.73	1	0	0	2	4	0
05	106.33	352.364	2.81	1	0	0	2	4	0
06	100.54	327.311	3.03	0	0	0	2	4	0
07	82.54	315.343	3.43	1	0	0	1	4	0
08	82.54	341.381	3.50	1	0	0	1	4	0
09	76.74	316.328	3.73	0	0	1	0	4	0
10	82.54	343.397	4.27	1	0	0	1	4	0
11	82.54	369.434	4.29	1	0	0	1	4	0
12	99.61	371.407	3.61	1	0	0	1	5	0
Celecoxib	77.99	381.372	3.61	1	1	0	3	0	0
Diclofenac	49.33	296.152	4.57	0	1	0	1	2	0
Flurbiprofen	37.30	244.265	4.05	0	0	0	0	2	0
Indomethacin	68.54	357.793	3.99	0	0	0	1	4	0
Olaparib	86.37	434.469	2.52	0	1	0	4	3	0

**Figure 1:**
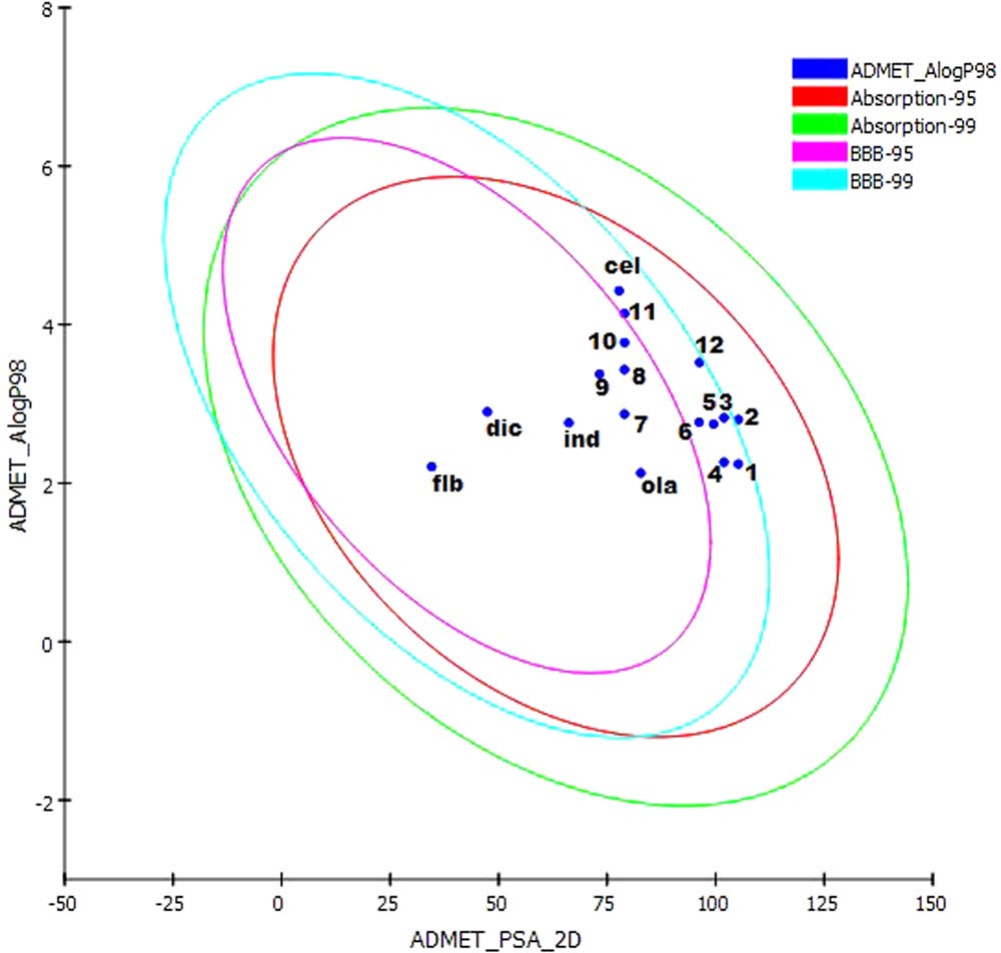
The plot of polar surface area (PSA) versus ALogP for designed thieno[3,2-c]pyran  analogs showing the 95% and 99% confidence limit ellipses corresponding to the blood-brain barrier (BBB) and intestinal absorption.

The calculated TPSA values of the designed compounds were within acceptable limits. Further, the distribution of compounds in the human body as indicated by blood-brain barrier coefficient (logBB), apparent Caco-2 permeability, log Kp for skin permeability, the volume of distribution, and plasma protein binding (log Khsa for serum protein binding) were determined to be within the standard range^48,49^. All compounds except **3** and **6** possessed good aqueous solubility (Table S1, Fig. 1), while compounds **1, 2, 4**, and **5** showed medium apparent MDCK permeability. The calculated logS values of the analogs were within the acceptable interval, and all analogs showed compliance with a standard range of ADME (Table 2). The analogs were comparable to reference drugs, celecoxib, diclofenac, flurbiprofen, and indomethacin. All the designed compounds were within their permissible limits and hence are unlikely to have limited bioavailability.

**Table 2:** Compliance of designed thieno[3,2-c]pyran analogues and standard drugs of computational parameters for pharmacokinetics (ADME).

Compounds	log Khsa for Serum Protein Binding	log BB for brain/blood	No. of metabolic reactions	Predicted CNS Activity	log HERG for K + Channel Blockage	Apparent Caco-2 Permeability (nm/sec)	Apparent MDCK Permeability (nm/sec)	log Kp for skin permeability	% Human Oral Absorption in GI ( + −20%)	Qual. Model for Human Oral Absorption
01	0.141	−1.399	1	−2	−6.438	21.792	14.4	−6.632	60.632	Medium
02	0.301	−1.437	3	−2	−6.214	21.503	12.65	−6.829	62.161	Medium
03	−0.062	−1.761	2	−2	−5.725	94.397	63.709	−4.499	74.929	HIGH
04	−0.012	−1.424	1	−2	−6.246	16.134	10.399	−6.783	55.551	Medium
05	0.141	−1.447	3	−2	−5.979	16.036	9.216	−6.981	57.051	Medium
06	−0.22	−1.785	2	−2	−5.532	70.311	46.344	−4.644	70.243	high
07	0.31	−0.627	2	1	−6.073	77.715	56.875	−5.626	74	High
08	0.465	−0.649	4	1	−5.792	77.204	50.364	−5.828	75.486	High
09	0.208	−0.985	3	−1	−5.343	338.668	253.487	−3.49	88.938	High
10	0.548	−0.747	3	0	−5.954	76.556	49.386	−5.719	77.164	High
11	0.707	−0.756	4	0	−5.881	77.473	50.566	−5.813	79.28	High
12	0.197	−1.463	4	−2	−5.355	168.045	101.097	−4.158	80.447	High
Celecoxib	0.367	−0.781	1	−1	−5.772	356.195	790.105	−3.223	92.188	High
Diclofenac	0.034	−0.163	4	−1	−2.892	384.134	809.104	−1.785	100	High
Flurbiprofen	0.163	−0.342	1	−1	−3.143	339.99	298.2	−1.948	96.585	High
Indomethacin	0.062	−0.608	3	−1	−3.243	187.398	253.764	−2.565	92.548	High
Olaparib	0.104	−1.188	1	−2	−4.182	196.841	207.7	−3.287	84.563	High
Stand. Range*	(−1.5/1.5)	(−3.0/1.2)	(1.0/8.0)	−2 (inactive) + 2 active)	(concern below −5)	(<25 poor, > 500 great)	(<25 poor, > 500 great)	(–8.0 to –1.0, Kp in cm/hr)	(<25% is poor)	(>80% is high)

### Compliance with toxicity risk assessment parameters

The toxicity risk assessment for all of the designed compounds and standard drugs were calculated using TOPKAT software by Discovery Studio 3.5 (Accelrys, USA). The toxicity risk parameter includes reproductive/developmental toxicity, TOPKAT Ames mutagenicity, TOPKAT skin irritant effects, TOPKAT ames score, TOPKAT rat oral LD50 (mg/kg), and TOPKAT rat carcinogenic potency TD50 (mg/kg) (Table 3). The TOPKAT computes probable toxicity values for a chemical structure by calculating a discriminant score based on its QSTR model. The discriminant score of all the compounds was found to be negative, indicating their non-carcinogenic property. The majority of the compounds were non-toxic for reproductive/developmental toxicity except for compounds **1, 3, 4, 6, 9**, and **12**. Likewise compounds **4** and **7** were mutagenic and compounds **9** and **12** showed skin irritability; these compounds were in compliance with drug-like parameters and comparable with the predicted results of the reference compounds celecoxib, diclofenac, flurbiprofen, indomethacin, and olaparib. Other designed compounds that showed toxicity still need to be further optimized.

**Table 3:** Compliance of designed thieno[3,2-c]pyran analogues and standard drugs to computational toxicity risks parameters (i.e., mutagenicity, tumorigenicity, irritation, and reproduction).

Compounds	Reproductive effects	TOPKAT Ames Mutagenicity	TOPKAT Skin Irritancy	TOPKAT Ames Score	TOPKAT Rat Oral LD_50_ (mg/kg)	TOPKAT Rat Carcinogenic potency TD_50_ (mg/kg)
01	Toxic	Non-Mutagen	Non-Irritant	−3.6508	2.6589	15.3886
02	Non-Toxic	Non-Mutagen	Non-Irritant	−3.2304	3.9341	4.7794
03	Toxic	Non-Mutagen	Non-Irritant	−4.8779	0.5851	32.7399
04	Toxic	Mutagen	Non-Irritant	−0.5579	1.3455	6.7780
05	Non-Toxic	Non-Mutagen	Non-Irritant	−1.1059	2.0047	2.1199
06	Toxic	Non-Mutagen	Non-Irritant	−1.7774	0.1696	14.4274
07	Non-Toxic	Mutagen	Non-Irritant	−0.3380	3.6710	10.3481
08	Non-Toxic	Non-Mutagen	Non-Irritant	−1.4741	5.7619	3.2442
09	Toxic	Non-Mutagen	Irritant	−1.5381	0.8082	22.0288
10	Non-Toxic	Non-Mutagen	Non-Irritant	−2.0154	2.0326	9.6870
11	Non-Toxic	Non-Mutagen	Non-Irritant	−3.3643	1.9045	3.0804
12	Toxic	Non-Mutagen	Irritant	−3.9004	0.9968	7.8474
Celecoxib	Non-Toxic	Non-Mutagen	Non-Irritant	−10.4112	4.0652	3.3486
Diclofenac	Non-Toxic	Non-Mutagen	Non-Irritant	−10.8461	0.6185	61.8037
Flurbiprofen	Non-Toxic	Non-Mutagen	Irritant	−4.9857	0.5641	44.0478
Indomethacin	Non-Toxic	Non-Mutagen	Non-Irritant	−18.4359	0.2095	4.7492
Olaparib	Non-Toxic	Non-Mutagen	Non-Irritant	−10.3633	0.8587	1.6315

### Binding affinity by molecular docking

In the present study, we explored the interactions and binding affinities of designed thieno[3,2-c]pyran analogs, RONS, and the reference drugs olaparib^50^, celecoxib, and flurbiprofen with the protein targets SIRT6 and COX-2. The binding affinities were determined as the SYBYL-X ‘total’ docking score.

#### Molecular Docking Analysis of SIRT6

**Figure 2:**
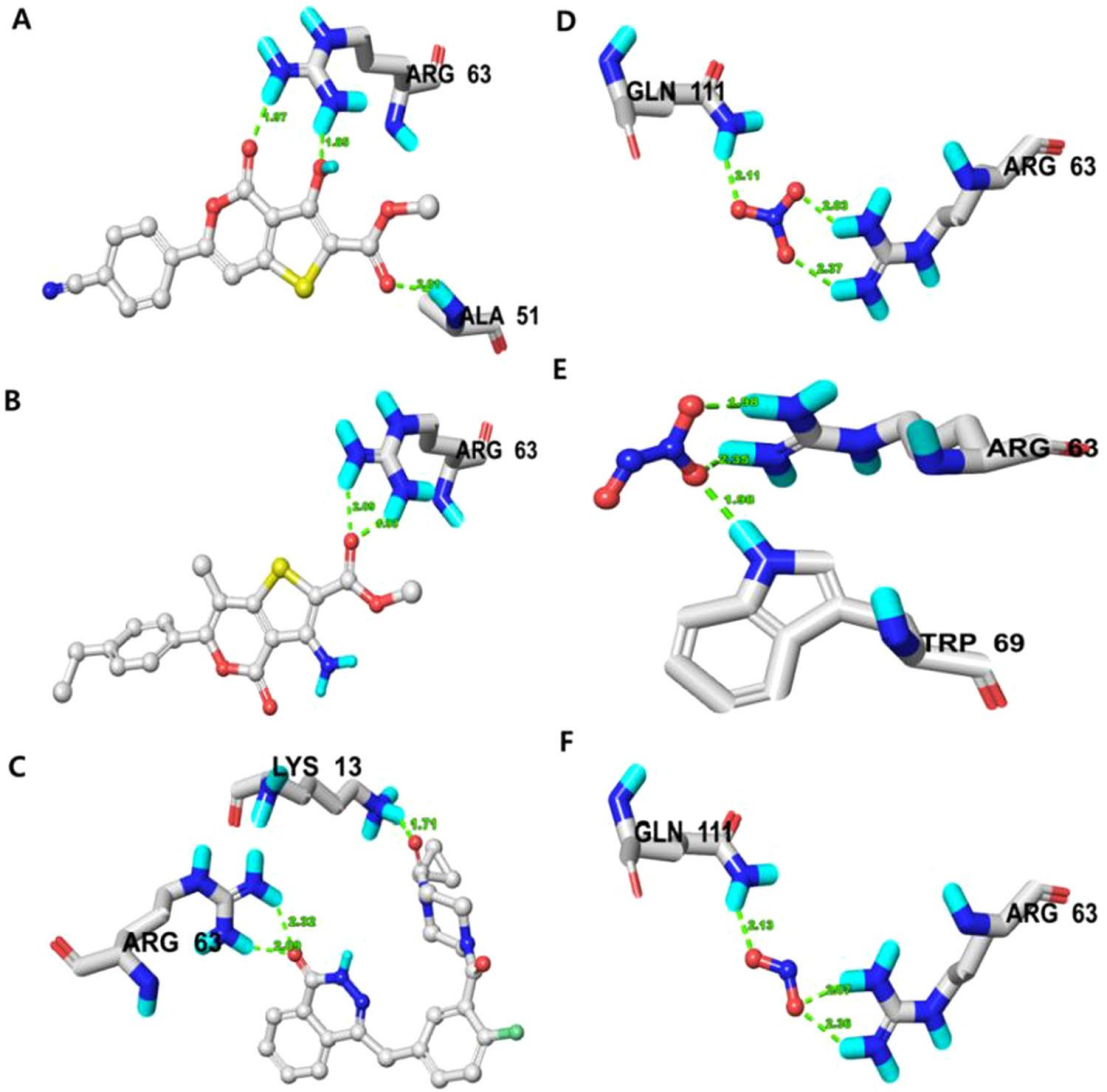
(**a**) Docked complex of compound 06, (**b**) compound 10, (**c**) Olaparib, (**d**) NO_3_ spe-cies, (**e**) N_2_O_3_ species and (**f**) NO_2_ species on SIRT6 (PDB ID: 3K35) revealing respective binding amino acid residues. Residues showing H-bond within 3 Å is displayed.

A docking simulation was performed in order to investigate the binding affinity of thieno[3,2-c]pyran analogs and RONS to SIRT6 (PDB ID: 3K35) protein. The docked poses of thieno[3,2-c]pyran analogs were ranked using the surflex-dock score and top score poses were selected for further analysis (Tables 4 and S2). The docking results of compound **6** and SIRT6 showed a high binding affinity represented by a high docking score (6.0956) relative to thieno[3,2-c]pyran analogs. Compound **6** forms three H-bonds (1.8, 1.9, 2.0 Å) to the polar, basic, positively charged nucleophilic residue made up of arginine (Arg)-63 and the backbone atom of alanine (Ala)-51 (Fig. 2a). In the docking pose, the binding site residues on SIRT6 within a radius of 3 Å from the bound compound were polar and uncharged. These residues consisted of the aliphatic, hydrophobic threonine (Thr)-213, glycine (Gly)-50, Ala-51, and isoleucine (Ile)-183, Ile-217; the polar and positively charged Arg-63, the aromatic, hydrophobic phenylalanine (Phe)-62 and trytophan (Trp)-186, the polar, uncharged, and nucleophilic serine (Ser)-214, the polar amide glutamine (Gln)-111, and the polar, acidic aspartic acid (Asp)-185.

**Table 4:** Comparison of binding affinity of selected designed thieno[3,2-c] pyran analogues, standard drugs and RONS against SIRT6 (PDB ID: 3K35) enzyme.

Compounds/RONS	Total Score	Amino acids in the binding site within 3.0 Å of ligand (H-bonding residues shown in bold)	Length of H-bond Å	No. of H-Bond
6	6.0956	Gly-50, **Ala-51**, Phe-62, **Arg-63**, Gln-111, Ile-183, Asp-185, Trp-186, Thr-213, Ser-214, Ile-217	1.85, 1.97, 2.01	3
10	6.0431	Phe-62, **Arg-63**, Trp-69, Gln-111, Met-155, Lys-158, Gly-156, Leu-184, Asp-185, Trp-186, Ile-217	1.93, 2.09	2
Olaparib	8.3569	**Lys-13**, Ala-51, **Arg-63**, Trp-69, Asn-112, Val-113, His-131, Ile-183, Leu-184, Asp-185, Trp-186	1.71, 2.09, 2.32	3
NO_3_	5.2070	**Arg-63, Gln-111**, His-131, Ile-217	2.37, 2.03, 2.11	3
N_2_O_3_	4.6042	Phe-62, **Arg-63, Trp-69**	2.35, 1.98, 1.98	3
NO_2_	4.3973	**Arg-63, Gln-111**, His-131, Ile-217	2.36, 2.07, 2.13	3

Note: Surflex-Dock scores (total scores) were expressed in - log_10_ (Kd) units to represent binding affinities.

Similarly, docking results for compound **10** with SIRT6 showed a good binding affinity (6.0431) and H-bond lengths of 1.9 Å and 2.0 Å to the nucleophilic (polar, basic, positively charged) residue, Arg-63 (Fig. 2b). In the docking pose, the chemical nature of the binding site residues within a radius of 3 Å from the bound compound were polar amide (Gln-111), polar-acidic (Asp-185), aromatic-hydrophobic (Phe-62, Trp-69, Trp-186), hydrophobic-aliphatic(Gly-156, Leu-184, Ile-217, and methionie (Met)-155, polar, positively charged (Lys-158). The docking score for all the thieno[3,2-c]pyran analogs ranged from 4.8363–6.0956 and a careful analysis of all binding poses revealed that the stability of thieno[3,2-c]pyran analogs has been implicated by hydrophobic and hydrophilic amino acid residues, however, hydrophobic amino acid residues favor stability and activity.

Furthermore, the binding interaction and binding affinities of thieno[3,2-c]pyran analogs were compared with those of olaparib, an FDA approved targeted therapy for cancer. The docking results for olaparib onto SIRT6 showed a high binding affinity indicated by docking score of 8.3569, formed by three H-bonds of lengths 1.7, 2.0, and 2.3 Å to nucleophilic, polar, basic, positively charged residues (Arg-63 and Lys-13). In the docking pose, the chemical nature of binding site residues within a radius of 3 Å from bound olaparib was basic, polar, positively charged (Arg-63, His-131, Lys-13), aliphatic-hydrophobic (Ile-183, Leu-184, Val-113, aromatic-hydrophobic (Trp-69, Trp-186, Phe-62), polar amide (Asn-112, Gln-111), and polar acid (Asp-185) (Fig. 2c). Analyzing the binding amino acid residues for olaparib reveals that it binds to hydrophobic and hydrophilic residues, where the hydrophobic residues stabilize in the binding pocket and the H-bond network is established by hydrophilic residues. Moreover, the thieno[3,2-c]pyran analogs and olaparib share the same amino acid residues that bind with the SIRT6 protein. Thus these thieno[3,2-c]pyran analogs could be a potential starting point that could be optimized into a cancer therapy drug targets.

Likewise, SIRT6 has been implicated in numerous types of cancer and RONS produce oxidative stress modulates the function of target proteins. Thus, RONS targeting of SIRT6 may alter SIRT6 function to inhibit cancer progression. In order to understand the interaction and binding affinities of RONS towards SIRT6, all RONS were docked into the SIRT6 binding pocket and ranked using the surflex-dock score. The three RONS molecules with the top scores were selected for further discussion where NO_3_ emerged as the RONS species with the strongest binding affinity to SIRT6.

The docking results for NO_3_ showed good binding affinity to SIRT6, as indicated by a docking score of 5.2070. NO_3_ forms three H-bonds with SIRT6 with lengths of 2.3, 2.0, and 2.1 Å. These bonds bind to a basic, polar, positively charged residue (Arg-63) and a polar amide (Gln-111). In the docking pose, the chemical nature of binding site residues within a radius of 3 Å from bound NO_3_ was basic polar, positively charged (Arg-63), polar uncharged (Gln-111), nucleophilic basic, polar (His-131), and aliphatic-hydrophobic (Ile-217) (Fig. 2d). Similarly, N_2_O_3_ and NO_2_ showed comparable binding affinities, indicated by the docking scores of 4.6042 and 4.3973, respectively. N_2_O_3_ forms three H-bonds of lengths 2.3, 1.9 and 1.9 Å to a basic polar, positively charged residue (Arg-63) and an N-H moiety of an aromatic hydrophobic residue (Trp-69) (Fig. 2e). Moreover, NO_2_ forms three H-bonds of lengths 2.3, 2.0, and 2.1 Å to basic polar, positive charged residues (Arg-63) and polar uncharged residues (Gln-111) (Fig. 2f). In the docking pose, the chemical nature of the binding site residues was basic polar, positively charged (Arg-63, His-131), aromatic hydrophobic (Phe-62, Trp-69) and aliphatic hydrophobic (Ile-217), and polar uncharged (Gln-111). When comparing the amino acid residues for all RONS species that bound to SIRT6, majority of the binding site residues were hydrophilic and were able to modulate the function of the target protein.

#### Molecular Docking Analysis of Designed Analogies into COX-2

**Figure 3:**
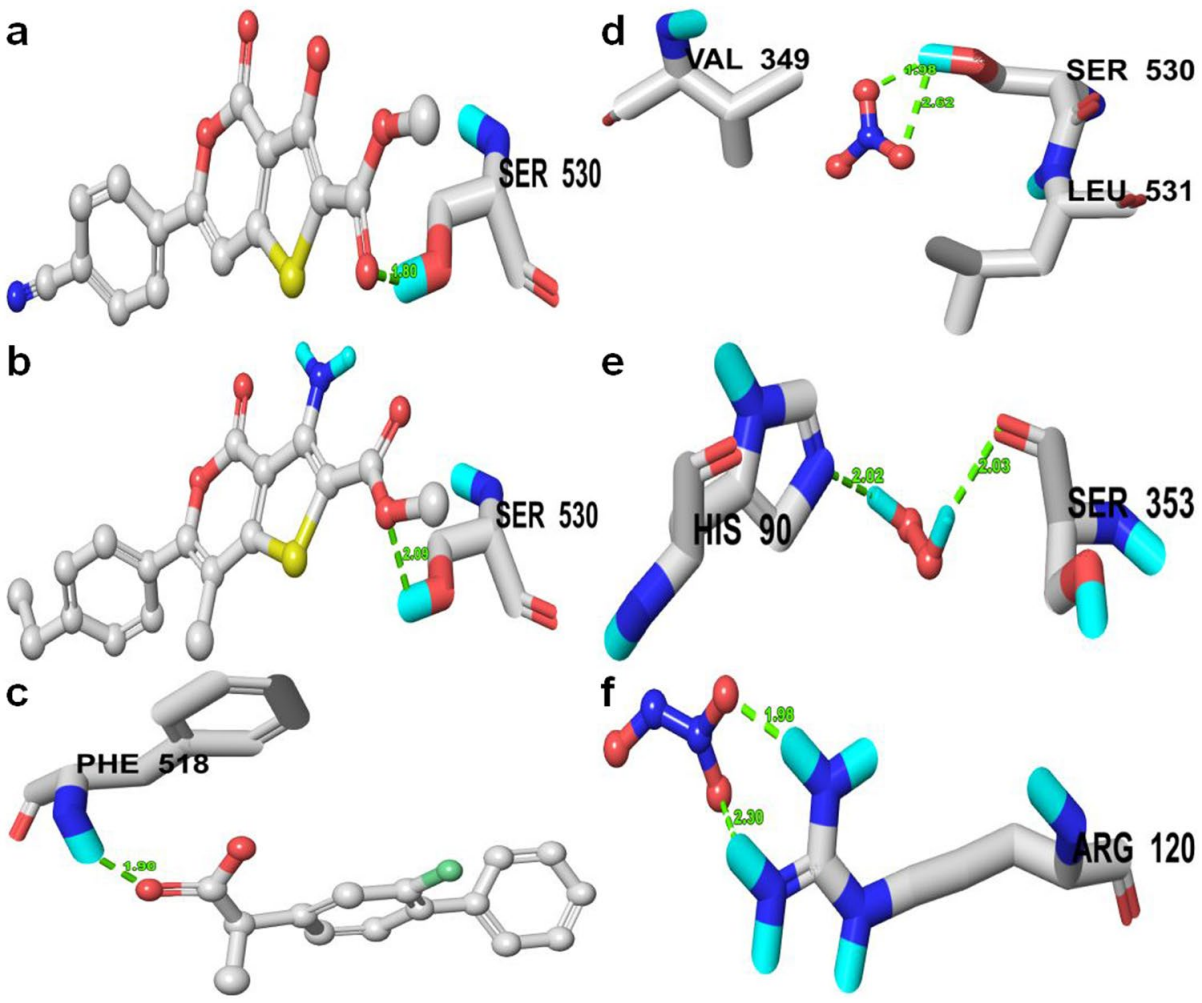
Docked complex of (**A**) Compound 06, (**B**) Compound 10, (**C**) Olaparib, (**D**) NO_3_, (**E**) N_2_O_3_ and (**F**) NO_2_ on anti-inflammatory target COX-2 (PDB: 6COX), residue showing H-bond within 3 Å is displayed.

Similarly, all the designed thieno[3,2-c]pyran analogs were docked into the binding pocket of COX-2 to check the anti-inflammatory properties of the designed analogs. The molecular docking results exhibited good binding affinities with the target protein (PDB ID: 6COX) (Table S3). The majority of the compounds interact with active amino acids residues such as His-90, Gln-192, Tyr-348, Val-349, Leu-352, Ser-353, Tyr-385, Ala-516, Ile-517, Phe-518, and Ser-530 in the COX-2 receptor. Compounds with good binding affinities are shown in Tables 5 and S3 compared with standard drugs. Compound **6** possesses a high docking score of 6.8541 with an H-bond of length 1.7 Å to the polar uncharged residue (Ser-530) (Fig. 3a). Similarly, compound **10** possessed a docking score of 6.8841 with an H-bond length of 1.9 Å to the same polar uncharged residue (Ser-530) (Fig. 3b). The standard drugs celecoxib and flurbiprofen were also docked into the same binding pocket in order to compare their binding orientation and interaction with those of the thieno[3,2-c]pyran analogs. Celecoxib and flurbiprofin had total docking scores of 6.1641 and 6.3151, respectively. Flurbiprofen forms a H-bond length 1.8 Å to the backbone atom of Phe-518, while most of the designed compounds formed H-bonds with the polar uncharged residue (Ser-530) (Fig. 3c). In all the docking poses, the chemical nature of the residues at the binding sites within 3 Å from bound ligands were polar positively charged residue (His-90), polar uncharged residues (Gln-192, Ser-353, Ser-530); aromatic hydrophobic residues (Tyr-355, Tyr-385, Phe-381, Phe-518, Trp-387) and aliphatic hydrophobic residues (Val-349, Val-523, Leu-352, Leu-534, Ala-516, Ile-517. Comparing the binding affinities of compounds **6** and **10** with COX-2 effectively and bind to the same amino acid residues as standard drugs. The docking outcomes suggest that these analogs ought to act as a potential scaffold for the design of inhibitors for inflammation-induced COX-2.

**Table 5:** Comparison of binding affinity of selected designed thieno[3,2-c] pyran analogues, standard drugs and RONS against the COX-2 (PDB ID: 6COX) enzyme.

Compounds/RONS	Total Score	Amino acids in the binding site within 3.0 Å of ligand (H-bonding residues shown in bold)	Length of H-bond Å	No. of H-Bond
6	6.8541	His-90, Val-349, Leu-352, Ser-353, Tyr-355, Phe-381, Tyr-385, Trp-387, Ala-516, Ile-517, Phe-518, Val-523, **Ser-530**, Leu-534	1.7	1
10	6.8841	His-90, Val-344, Val-349, Leu-352, Ser-353, Tyr-355, Phe-381, Leu-384, Tyr-385, Trp-387, Arg-513, Ala-516, Ile-517, Phe-518, Met-522, Val-523, Gly-526, **Ser-530**, Leu-534	1.9	1
Flurbiprofen	6.1641	His-90, Gln-192, Val-349, Leu-352, Ser-353, Tyr-355, Arg-513, Ala-516, Ile-517, **Phe-518**, Val-523, Ala-527, Leu-531	1.8	1
Celecoxib	6.3151	Met-113, Val-116, Trp-348, Val-349, Leu-352, Ser-353, Tyr-355, Leu-359, Tyr-385, Val-523, Gly-526, Ala-527, Ser-530, Leu-531	—	—
NO_3_	3.2150	Val-349, Leu-531, **Ser-530**	2.62, 2.60, 1.98	3
H_2_O_2_	3.1340	Tyr-355, **Ser-353, His-90**	2.02, 2.03	2
N_2_O_3_	3.0906	**Arg-120**, Ala-527, Val-523, Ser-353, Tyr-355	1.98, 2.30	2

Note: Surflex-Dock scores (total scores) were expressed in - log 10 (Kd) units to represent binding affinities.

Apart from the docking of the designed thieno[3,2-c]pyran analogs, we also studied the interaction of RONS with COX-2 and observed how RONS interact with amino acid residues at the binding site of COX-2. Thus, all RONS, such as H_2_O_2_, NO_2_, N_2_O, NO_3_, N_2_O_3_, and N_2_O_5_ were docked into the binding pocket of COX-2 and given a surflex-dock score. From the docking results, the binding interactions of the top three RONS species were discussed. Apparently, among the top-ranked docked RONS species, NO_3_ was found to possess good binding affinity as indicated by total docking score of 3.2150, along with a higher binding affinity for SIRT6 protein. In the docking pose, the chemical natures of the binding site residues within a radius of 3 Å from bound NO_3_ were nonpolar, aliphatic, and hydrophobic (Leu-531, Val-349). The docked pose of NO_3_ to COX-2 includes three H-bonds to nucleophilic polar, uncharged residues (Ser-530) (Fig. 3d). Similarly, docking results for H_2_O_2_ species showed a docking score of 3.1340 and the formation of two H-bonds of length 2.0 Å to the nucleophilic polar, uncharged residue (Ser-353), and basic (polar) residue (His-131). Tyr-355, an aromatic hydrophobic amino acid residue was also found within a radius of 3 Å (Fig. 3e). Furthermore, the docking results of N_2_O_3_ showed a strong binding affinity to COX-2, as shown in the docking score of 3.0906, and the formation of two H-bonds of lengths 1.9 and 2.3 Å to a nucleophilic polar, basic, positively charged residue (Arg-120) (Fig. 3f). In the docking pose, the chemical natures of the residues were hydrophobic (Ala-527, Val-523), nucleophilic, polar, uncharged (Ser-353), and aromatic hydrophobic (Tyr-355). The amino acid residues in COX-2 involved in binding of all docked RONS species were mainly hydrophilic in nature, thus it may be pointed out that all RONS species target hydrophilic amino acid residues in order to modulate the function of target proteins.

The docking results and analysis of all RONS poses when bound to SIRT6 or COX-2 revealed that hydrophilic residues play an important role in modulating the function of the target proteins. In addition, studies of the interaction of RONS with protein/lipid/membrane system, RONS also reveal skin cancer targets and hydrophilic/double bond-containing substituents on the protein/lipid membrane system to modulate the functions of the system^51–53^.

### Molecular Dynamic Simulations for thieno[3,2-c]pyran analogs

The stability of the complex system in an aqueous solution was examined using the parameters RMSD (root mean square deviation), RMSFs (root means square fluctuations), and radius of gyration (Rg). For this simulation, a 10 ns unconstrained simulation was performed on the docked structure of SIRT6 (3K35) and COX-2 (6COX) bound to compounds **6** and **10** and to the reference drugs olaparib, flurbiprofen, and celecoxib.

**Figure 4:**
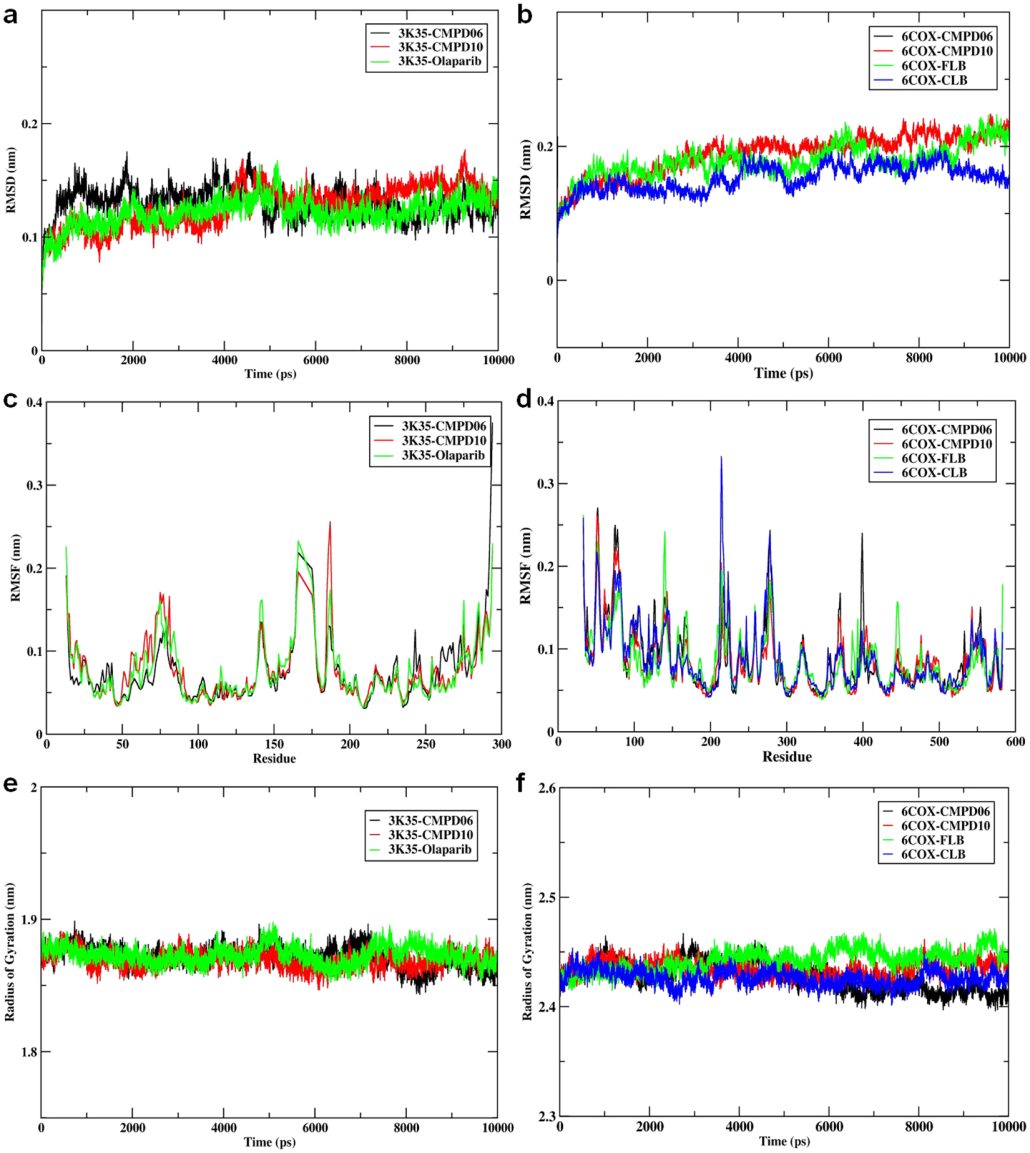
(**a**) Time dependence of root mean square deviations (RMSDs) for Compound06, Compound10, Olaparib in complex with 3K35 protein; (**b**) Time dependence of root mean square deviations (RMSDs) for Compound06, Compound10, Flurbiprofen, Celecoxib in com-plex with 6COX protein; (**c**) Root mean square fluctuations (RMSF) for Compound06, Com-pound10, Olaparib in complex with 3K35 protein; (**d**) Root mean square fluctuations (RMSF) for Compound06, Compound10, Flurbiprofen, Celecoxib in complex with 6COX protein; (**e**) Time evolution of the radius of gyration (Rg) for Compound06, Compound10, Olaparib in complex with 3K35 protein (**f**) Time evolution of the radius of gyration (Rg) for Compound06, Compound10, Flurbiprofen, Celecoxib in complex with 6COX protein during 10000 ps mo-lecular dynamics (MD) simulation.

Despite the initial structural arrangements of the docked complex, the average RMSD of the trajectories for bound protein backbone atoms showed relative stability. Figure 4a,b display the stable RMSD values of the atoms for docked compounds 3K35 and 6COX proteins respectively. The RMSD analysis for 3K35 and 6COX indicates that they reach equilibration and oscillate around an average value after 1000 ps. The average RMSDs from 1000 ps to 10000 ps for compounds **6** and **10** and olaparib bound to 3K35 protein were 0.13 nm, 0.13 nm, and 0.12 nm, while the values for compounds **6** and **10**, celecoxib, and flurbiprofen bound to 6COX were 0.17 nm, 0.19 nm, 0.16 nm, and 0.18 nm, respectively.

These RMSD results show the relative stability of compounds **6** and **10** and olaparib bound to 3K35 throughout the simulation, while for 6COX, compound **10** and flurbiprofen had slightly higher RMSDs than compound **6** and celecoxib. This result suggests that compound **10** and flurbiprofen undergo a structural conformational change during the simulations.

Local protein mobility was analyzed by measuring the time-averaged RMSF values of selected compounds bound to 3K35 and 6COX proteins against residue numbers based on 10000 ps trajectory data. The average RMSFs measured for compounds bound to 3K35 were 0.074 nm (compound **6**), 0.077 nm (compound **10**), and 0.075 nm (olaparib), which reveals the relative stability of the complex upon binding (Fig. 4c). Further, the average atomic fluctuations of compounds bound to 6COX protein were also measured and found to be 0.088 nm (compound **6**), 0.084 nm (compound **10**), 0.088 nm (celecoxib), and 0.085 nm (flurbiprofen) (Fig. 4d). Moreover, comparing the RMSFs for both bound 3K35 and 6COX proteins near the binding site regions suggest that all the designed compounds show similar binding patterns profiles for their respective target proteins and stabilize them in their favorable conformations for inhibition.

We also determined the Rg value, which provides insight into the overall dimension and the shape of the protein. Figure 4e,f display Rg values for 3K35 and 6COX proteins in bound form, respectively. The average Rg values were 1.87 nm (compound **6**), 1.86 nm (compound **10**), 1.87 nm (olaparib) for 3K35 and 2.43 nm (compound **6**), 2.43 nm (compound **10**), 2.44 nm (celecoxib), and 2.43 nm (flurbiprofen) for 6COX protein. The results of the average Rg values indicate that overall shape of the protein was stable upon binding of the ligand.

### Molecular Dynamic Simulations for RONS

**Figure 5:**
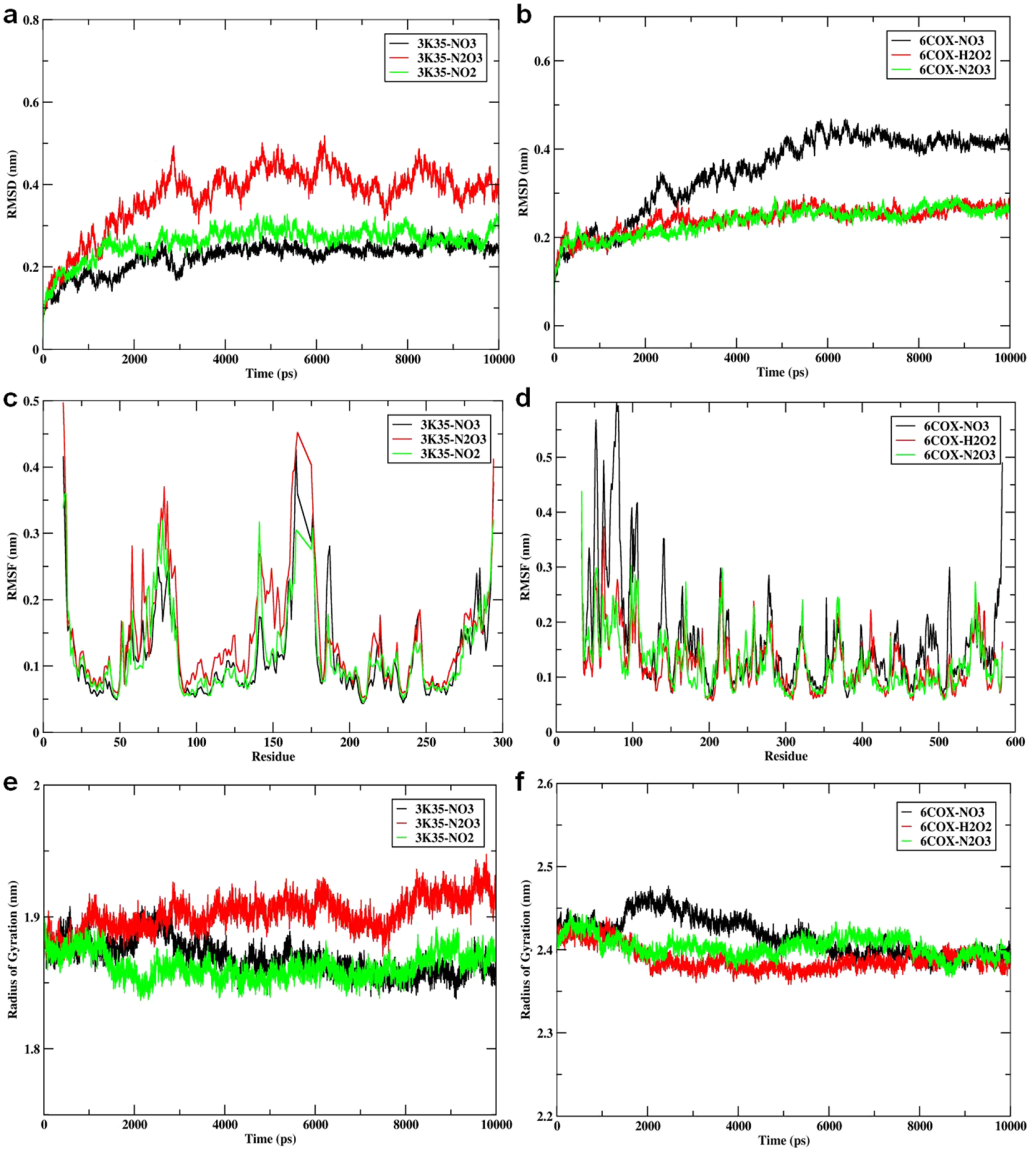
(**a**) Time dependence of root mean square deviations (RMSDs) for NO_3_, N_2_O_3_, NO2 in complex with 3K35 protein; (**b**) Time dependence of root mean square deviations (RMSDs) for NO_3_, H_2_O_2_, N_2_O_3_ in complex with 6COX protein; (**c**) Root mean square fluctuations (RMSF) for NO_3_, N_2_O_3_, NO_2_ in complex with 3K35 protein; (**d**) Root mean square fluc-tuations (RMSF) for NO_3_, H_2_O_2_, N_2_O_3_ in complex with 6COX protein; (**e**) Time evolution of the radius of gyration (Rg) for NO_3_, N_2_O_3_, NO_2_ in complex with 3K35 protein (**f**) Time evolution of the radius of gyration (Rg) for NO_3_, H_2_O_2_, N_2_O_3_ in complex with 6COX protein during 10000 ps molecular dynamics (MD) simulation.

We further investigated the 3K35 and 6COX proteins system in an aqueous environment supplemented with RONS. The properties studied, including the RMSD, RMSF, Rg, and mean square displacement (MSD), were acquired by performing a 10 ns unconstrained simulation of the docked complex structures of 3K35 and 6COX with top binding poses of NO_3_, N_2_O_3_, NO_2,_ NO_3_, H_2_O_2_, and N_2_O_3_, respectively. Despite being made up of tiny molecules, RONS significantly affect the structural properties of proteins. The average RMSD of the trajectories for RONS bound to 3K35 protein backbone atoms (Fig. 5a) shows that the bound complex of NO_3_ and NO_2_ achieved equilibrium after 2000 ps. Thereafter, the complexes fluctuated with RMSD values of 0.24 nm and 0.25 nm, respectively. Equilibrium for N_2_O_3_ bound to SIRT6 occurred after 3000 ps, after which the complex fluctuated with an RMSD value of 0.40 nm, which is higher than that of NO_3_ or NO_2_. A similar RMSD pattern was observed for RONS bound to 6COX protein (Fig. 5b). Average RMSD values for the trajectories of H_2_O_2_ and N_2_O_3_ bound to 6COX protein backbone atoms became stable after 4000 ps, and oscillated afterwards with an average value of 0.26 nm for both complexes. In contrast, large conformational changes were observed when NO_3_ bound to 6COX; the system was stable after 5000 ps and oscillate afterwards with an average length of 0.42 nm. The average RMSD for the whole frame was recorded with values of 0.34 nm, 0.24 nm, 0.24 nm for NO_3_, H_2_O_2_, and N_2_O_3_, respectively. Furthermore, the RMSD results for backbone atoms in both complex systems suggest that NO_3_ and NO_2_ for 3K35 and H_2_O_2_ and N_2_O_3_ for 6COX formed the most stable systems.

Similarly, the local binding effects of RONS towards amino acid chains of 3K35 and 6COX proteins were analyzed by measuring the time-averaged RMSF values and plotted against residue numbers based on 10000 ps trajectory data. The average atomic fluctuations for NO_3_, N_2_O_3_, and NO_2_ bound to 3K35 were 0.12 nm, 0.14 nm, and 0.12 nm, respectively (Fig. 5c), while the corresponding fluctuations for NO_3_, H_2_O_2_, and N_2_O_3_ bound to 6COX were 0.16 nm, 0.13 nm, and 0.13 nm, respectively (Fig. 5d). These results were all similar and comparable to each other, however, larger fluctuations were observed for the binding of NO_3_ to 6COX. Furthermore, a comparison of the RMSF results for the binding site regions of both 3K35 and 6COX proteins reveals that the amino acids involved in binding the ligand were stable and within their average values throughout the simulation, and that RONS exhibited similar binding patterns profiles.

Moreover, the globularity indices for 3K35 and 6COX proteins bound to RONS were measured by calculating Rg. Figure 5f display the Rg values for RONS bound to 3K35 and 6COX respectively. The average Rg values calculated for RONS bound to 3K35 were 1.86 nm (NO_3_), 1.90 nm (N_2_O_3_), and 1.86 nm (NO_2_), while those for 6COX were 2.41 nm (NO_3_), 2.38 nm (H_2_O_2_), and 2.40 nm (N_2_O_3_). The Rg indices for N_2_O_3_ bound to 3K35 and NO_3_ bound to 6COX were higher than the Rg indices for other RONS. A similar pattern was also seen for the RMSD and RMSFs values, which indicates that the binding of NO_3_ to either 3K35 or 6COX significantly affects the conformational behavior of the proteins and that NO_3_ could be an influential species for both target proteins.

**Figure 6:**
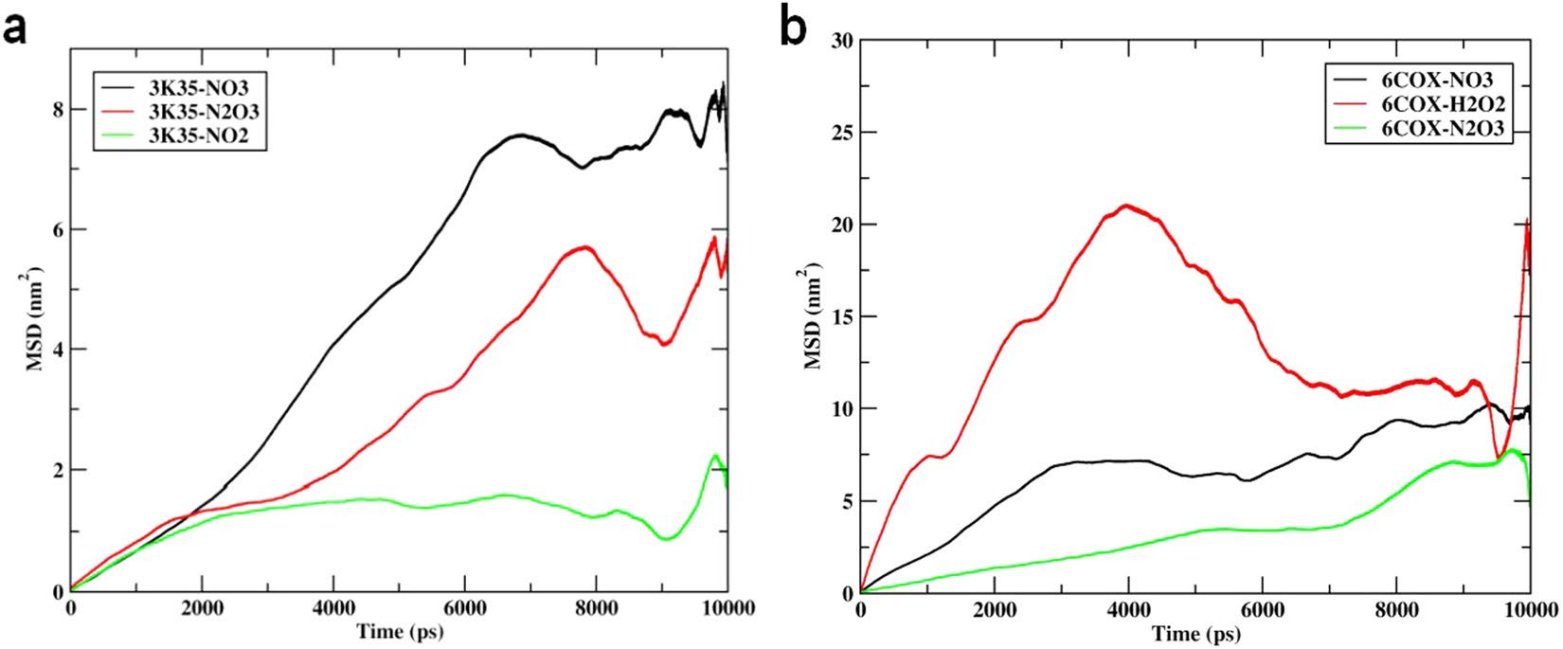
(**a**) Time dependence of mean square displacement (MSD) for NO_3_, N_2_O_3_, NO_2_ in complex with 3K35 protein; (**b**) mean square displacement (MSD) for NO_3_, H_2_O_2_, N_2_O_3_ in complex with 6COX protein during 10000 ps molecular dynamics (MD) simulation.

As mentioned above, since RONS are tiny in comparison to thieno[3,2-c]pyran analogs, we also investigated the diffusion coefficient of RONS in the binding pocket of 3K35 and 6COX by measuring the MSD values (Fig. 6a,b). The average MSD values for NO_3_, N_2_O_3_, and NO2 were calculated and found to be 0.1668 (±0.1232)cm^2^/s, 0.1059 (±0.0256)cm^2^/s, 0.0055 (±0.0471)cm^2^/s, respectively. The lower MSD values suggest the stability of RONS bound to 3K35 during the simulation. MSD values for NO_3_, H_2_O_2_, and N_2_O_3_ bound to 6COX were found to be 0.1047 (±0.0313)cm^2^/s, −0.0574 (±0.8425)cm^2^/s, and 0.1116 (±0.0646)cm^2^/s. Comparing the MSD values for RONS, a different pattern for H_2_O_2_ was observed, wherein the diffusion coefficient for H_2_O_2_ increases gradually, then decreases to the diffusibility level of NO_3_ and N_2_O_3_ species and then further increases. From these values, it appears that evidently H_2_O_2_, during their simulations, could have interacted with many atoms of protein residues, and consequently increased their diffusibility. However, the MSD values for NO_3_ and N_2_O_3_ species were normal and stable throughout their simulation.

The analysis of the molecular dynamic simulation revealed that NO_3_ and N_2_O_3_ were found to be particularly influential amongst all species of target proteins. Similarly, earlier reports on the interaction of RONS with proteins/lipid membrane systems also revealed that concentration-dependent RONS were known to modulate the functions of the target system.

### Metabolic Networks and Pathway Maps

**Figure 7:**
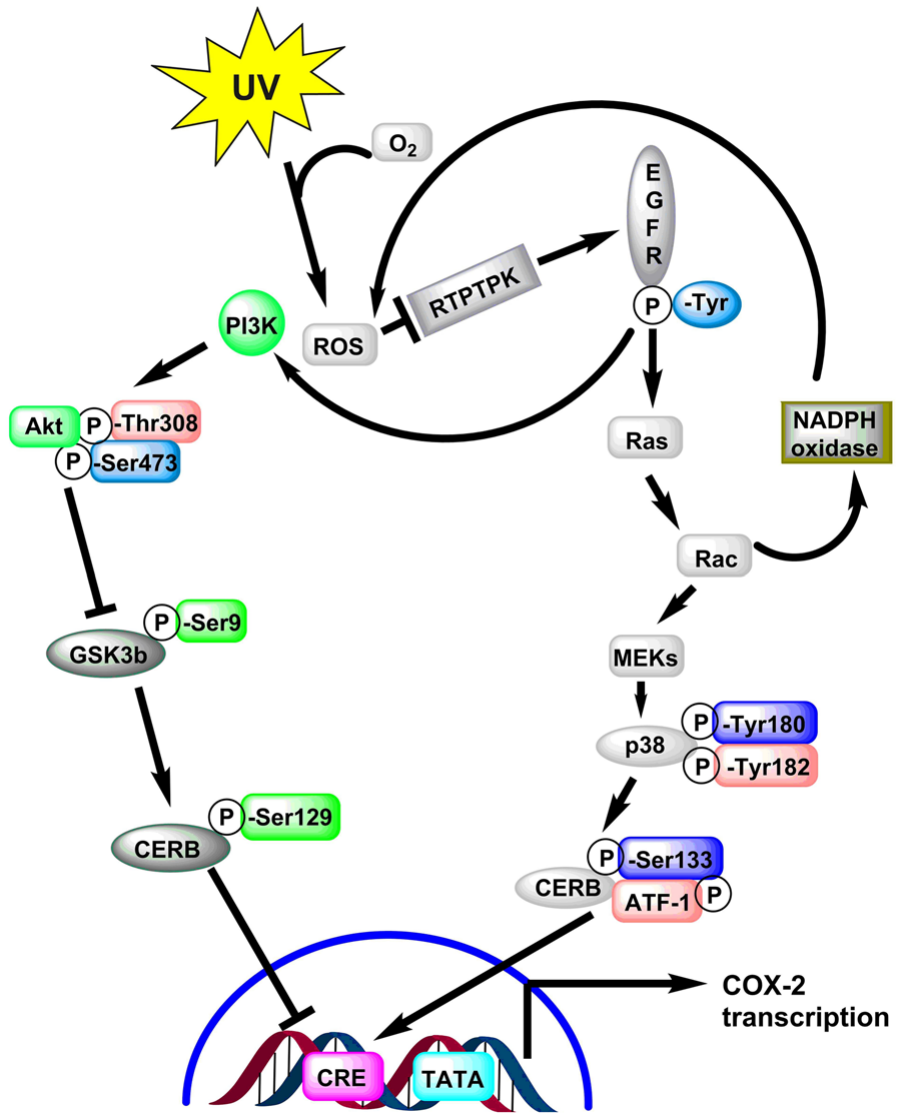
Molecular mechanisms by which UVB light induces cyclo-oxygenase-2 (COX-2) expression. Absorption of UV electromagnetic energy by molecules within a cell is transferred to molecular oxygen-generating ROS.

**Figure 8:**
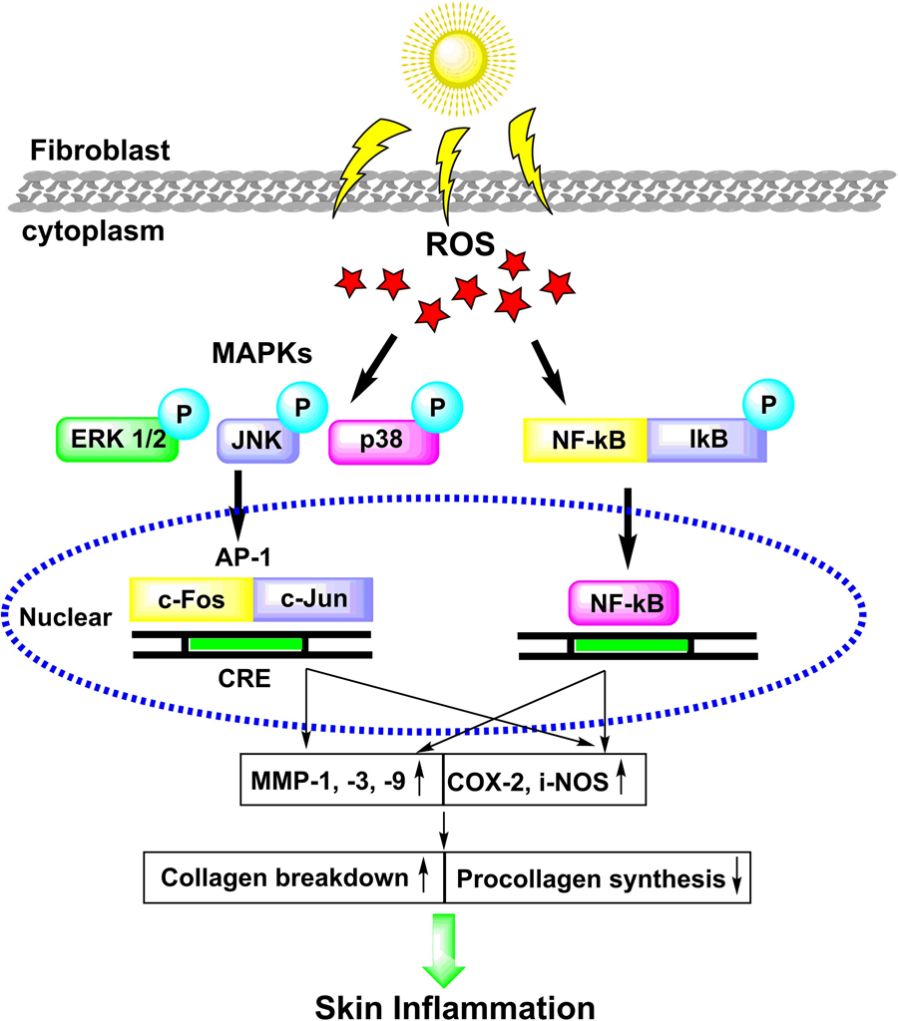
Schematic diagram showing effects of UVB induced in skin inflammatory and photo damage.

UV light energy is absorbed by molecules within the cell, and this energy is then transferred to molecular oxygen which uses it to produce ROS. Many of these ROS then react to form cysteine amino acids present in the active site of tyrosine phosphatases (Fig. 7) and MAP kinases (i.e. JNK, ERK, and p38) (Fig. 8). Specifically, tyrosine type protein receptor is inhibited by ROS, which is responsible for the inactive and phosphorylated state of epidermal growth factor receptor (EGFR)^54^. Therefore, inactivation of tyrosine type protein receptor phosphatase-κ by ROS leads to ligand-independent activation of EGFR, which in turn activates numerous downstream signaling pathways including the Ras⁄Rac1⁄p38 mitogen-activated protein kinase (MAPK) and phosphatidylinositol 3-kinase(PI3K)⁄Akt pathways^55,56^. Rac1 activation also induces additional ROS generation via the activation of NADPH oxidase^57^. p38 activation results in the phosphorylation of cyclic AMP response element (CRE) binding protein (CREB) and the activation of transcription factor-1, which then binds to the CRE site in the COX-2 gene promoter, activating transcription. PI3K activation by EGFR leads to phosphorylation (and thus activation) of Akt at both Thr-308 and Ser-473, which then in turn phosphorylates glycogen synthase kinase-3b (GSK-3β) at Ser-9, inactivating it. GSK-3β normally phosphorylates CREB at Ser-129, which is an inhibitory phosphorylation site, and thus inactivation of GSK-3β^53–55^ by Akt leads to dephosphorylation of CREB at Ser-129, relieving CREB inhibition^57–59^. This mechanism, in combination with the activating phosphorylation of CREB at Ser-133 by p38, is responsible for the binding of CRB at the CRE site on the COX-2 promoter. This binding, in turn, recruits CRB’s coactivator, CREB binding protein, which leads CRB to interact with the basal transcriptional machinery^57^. Several pathway inhibitors and overexpressing predominant negative specific kinases, both the PI3K/Akt and hep38 MAPK pathways have been shown to be necessary for the maximal induction of COX-2 expression by UVB^57–59^.

COX-2 and iNOS are enzymes that help mediate UV-induced inflammation^60^. UV-induced ROS drive the activation of MAP kinases (i.e., ERK, JNK, and p38), recruiting activator protein-1 (c-Fos and c-Jun) to the nucleus and, subsequently activating NF-κB and upregulating proinflammatory gene expression^61,62^. Another  reported study revealed that activator protein-1 and MAP kinase are responsible for regulating COX-2 expression^61^. It was reported that UV radiation causes nuclear translocation of NF-κB and hence induces the release of MMP in order to degrade the collagen in human skin^63,64^. Additionally, in another study, NF-KB modulated COX-2 and iNOS gene transcription and protein expression, causing skin inflammation^65,66^. Our in-silico hypothesis also supported this experimental work. The details of this pathway presented in Figs 7–8.

## Conclusion

UV-induced development of non-melanoma skin cancer (NMSCs) is the most prevalent existing cancer. The role of SIRT6 in the development of skin cancer is still debatable. However, previous experimental studies have reported that overexpressed SIRT6 results in increased inflammation, proliferation, and survival of sun-damaged skin cells. Likewise, COX-2 is also involved in the pathogenesis of UV-induced non-melanoma skin cancers, and its inhibition is presumed to prevent the development of NMSCs. The connection of SIRT6 and COX-2 has been widely determined; an increase in SIRT-6 expression leads to a greater abundance of COX-2 while inhibition of SIRT-6 expression leads to a decrease in SIRT-6 expression. The work presented here shows that chemical features of a set of designed compounds can be rationalized and that non-thermal cold atmospheric thermal plasmas (CAPs) can be utilized to modulate the functions of the enzyme. Herein, we report the design of thieno[3,2-c]pyran analogs, their binding to their targets, and the conformational changes they induce in the target proteins, using molecule docking and dynamics simulations. The molecular docking results of thieno[3,2-c]pyran analogs revealed that the active pocket of the protein target is hydrophobic in nature and can bind the molecules with hydrophobic groups. Hydrogen bonding features in the ligand also played an important role in their binding to the target protein. The pharmacokinetic compliance for the designed analogs was in agreement with the reference drugs. Moreover, they could be further optimized for lead/drug-like properties. The molecular docking and pharmacokinetic parameters suggested that compounds **6** and **10** possess good characteristics to be used as the lead-like molecule. The presence of a cyanide group and short alkyl group in the thieno[3,2-c]pyran scaffold could have caused the molecule to obtain the configuration necessary to bind to the target. The molecular dynamic studies revealed that compounds **6** and **10** bound to 3K35 and 6COX were stable and exhibited minimal conformational changes, compared to reference drugs. The effect of RONS (CAPs) was also studied within the binding pocket of the protein target and revealed the role of hydrophilic residues that modulating the structural properties of the target. Moreover, molecular dynamic studies revealed that NO_3_ and N_2_O_3_ influenced the conformational changes observed in the protein target. Thus, the present computational work reports the thieno[3,2-c]pyran scaffold for the development of analogs to targets SIRT6 and COX-2 enzymes, and highlights the role of CAPs in modulating the function of the enzyme.

## Materials and Methods

### Modeling Parameters and Geometry Optimization

**Figure 9:**
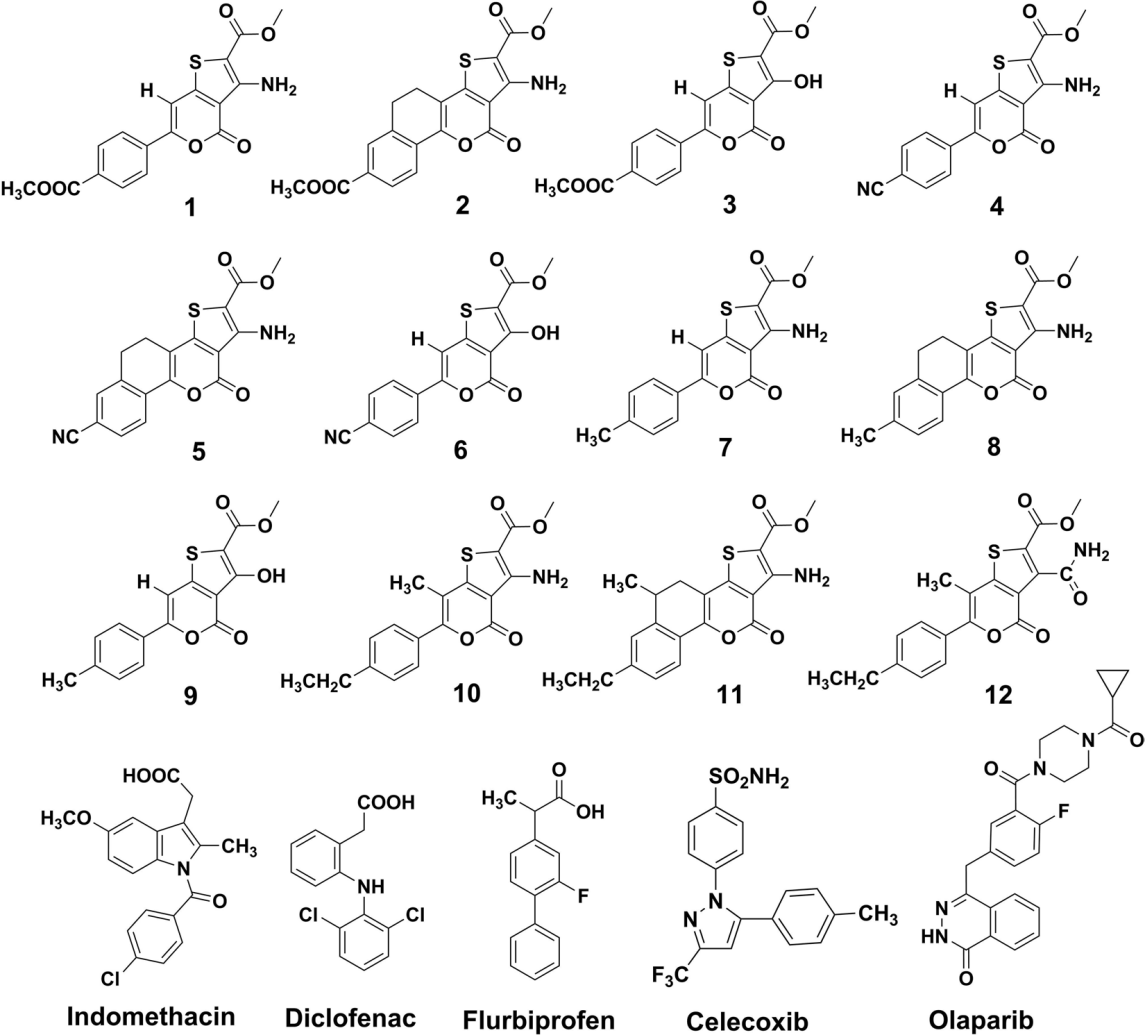
Structures of designed thieno[3,2-c]pyran analogues and reference drugs.

The structures of all the designed thieno[3,2-c]pyran analogs were drawn using ChemBioDraw Ultra v12.0 software (Fig. 9). The 2D structures were converted into 3D using the Concord program, followed by geometry cleaning and energy minimization performed by SYBYL-X 2.1.1. The Tripos force field^40,42,43,67^ and Gasteiger-Hückel charge were assigned during the energy minimization. The maximum iterations for the minimization were set to 2000 steps. The molecular structures of reactive oxygen and nitrogen species (RONS), including H_2_O_2_, NO_2_, N_2_O, NO_3_, N_2_O_3_, and N_2_O_5_ were built using the builder features of the maestro. To ensure the correct geometry and charges, a hybrid density functional theory (DFT) with Becke’s three-parameter exchange potential and the Lee-Yang-Parr correlation functional (B3LYP) and 6–31 G** basis was employed for QM-based geometry optimization and charge calculation^68^. Finally, these optimized structures were used to compute the properties of the RONS.

### Molecular Docking Experiment Parameters

To find the potential bioactive conformation of the thieno[3,2-c]pyran analogs, the SYBYL-X 2.1.1 suite with the Geom-X mode of the Surflex-Dock engine was used to perform the molecular docking simulation. Thieno[3,2-c]pyran analogs were used as ligands, and SIRT6 (PDB ID: 3K35)^69^ and COX-2 (PDB ID: 6COX)^70^ were used as a potential drug targets. The standard protocol was adopted for protein preparation before docking; i.e. addition of H-atoms, assignment of charges, the addition of missing side chains, removal of co-crystallized water molecules, and energy minimization^71,72^. The prepared structure was further used for the docking of thieno[3,2-c]pyran analogs and RONS. The surflex-dock scoring function was used to score the docking interaction. The surflex-dock score considers several factors related to ligand-receptor interaction, hydrophobicity, polarity, repulsiveness, entropy, and solvation. The docking parameters included ligand flexibility and rigid protein structure, and all other parameters were set to their default values. Furthermore, for RONS docking, the same binding pockets of 3K35 and 6COX along with QM-based charges generated from hybrid density functional theory (DFT).

### *In Silico* Pharmacokinetics Evaluation

Pharmacokinetic properties are described as the absorption, distribution, metabolism, excretion, and toxicity (ADMET) of a drug and provide key information about human therapeutic use of any compound. Pharmacokinetic data also includes information about drug solubility and the ability of the drug to be metabolized by cytochrome-P450 (CYP). This information may be used to determine the metabolism of possible future molecules. The metabolism of these predicted lead compounds was evaluated in detail by a plethora of diverse enzyme families that are involved in xenobiotic metabolisms, such as CYP450 enzymes, dehydrogenases, flavin-containing monooxygenases, hydrolases, peroxidases, UDP-glucuronosyl-transferases (UGTs), sulfotransferases, and glutathione S-transferases. The descriptors used herein correlate well with pharmacokinetic properties such as primary determinant of fractional absorption, and refer to the polar surface area (PSA) (cut-off ≤ 140 Å^2^), and low molecular weight (MW) for absorption^67,73^. For the secondary determinant of fractional absorption, we used the sum of H-bond donors and acceptors (cut-off ≤ 12); this descriptor was used to show passive membrane transport. The rotatable numbers of bonds number was used as a measure of flexibility (cut-off ≤ 10) and bioavailability. Drug distribution depends on several factors, including permeability (indicated by apparent Caco-2 and MDCK permeability, and log Kp for skin permeability), blood-brain barrier (log BBB), volume of distribution, and plasma protein binding (shown as logKhsa for serum protein binding)^48,49,74^. These ADME descriptors for the designed thieno[3,2-c]pyran analogs were calculated and checked for compliance with standard ranges. In addition, the octanol-water partition coefficient (log P) has been implicated in logBB penetration and permeability studies. The process of drug excretion from the body depends on log P and MW. Likewise, rapid renal clearance is associated with hydrophilicity and small molecules. In the liver, increased drug metabolism is associated with hydrophobicity and large molecules. Thus, higher lipophilicity leads to poor absorption and an increase in the metabolic process. The descriptors of 90% of orally active compounds follow Lipinski’s rule. Therefore, in order to ascertain the drug-like properties, all the parameters were calculated using QikProp v3.2 (Schrödinger, LLC, USA, 2015) and Discovery Studio 3.5 software^75^. Lastly, the toxicity parameter was evaluated by calculating different standard properties using TOPKAT software^76–79^.

### Molecular Dynamics Simulations

The molecular dynamics simulation was carried out for 10000 ps using the GROMACS 4.6.5 package^80^. The topology parameters for SIRT6 (3K35) and COX-2 (6COX) were generated by Gromacs, whereas for thieno[3,2-c]pyran analogs and RONS, the ACPYE module of AmberTools16^81^ and the Automated Topology Builder server^82^ were used, respectively. Charges for RONS, were adopted from QM-based hybrid DFT. Prior to simulation, an energy minimization was performed to full system without constraints using steepest descent integrator for 2000 steps. The system was then equilibrated for 200 ps of NVT and NPT ensemble, applying the position restraints on protein, inhibitors, and counterions at 310.15 K with periodic boundary conditions. The temperature was kept constant by a Berendsen thermostat, while the pressure was maintained at 1 bar using a Parrinello-Rahman scheme. Electrostatic interactions were calculated using the particle mesh Ewald method and cut-off distances for the calculation of Coulomb and van der Waals interactions were 1.4 nm during the equilibration. Finally, the system was subjected to 10000 ps MD at a temperature of 310.15 K (V-rescale thermostat) and a pressure of 1 bar (Parrinello-Rahman barostat). A periodic boundary condition was imposed on the system and the motion equations were integrated by applying the leaf-frog algorithm with a time step of 2 fs.

### Molecular Dynamic Simulation of SIRT6 and COX-2

The thieno[3,2-c]pyran analogs and RONS with the top docking scores were selected for a  10000 ps molecular dynamic simulation. The complex was placed in a cubic periodic box with a side length of 114.32 Å and the minimum distance between the complex and box walls was set to larger than 10 Å. The complex was comprised of 2736 atoms in the SIRT6 protein and 5835 atoms in the COX-2 protein. One inhibitor was solvated by single point charge (SPC) water molecules followed by the addition of counterions. The system was energy minimized and equilibrated prior to the molecular dynamic simulation run. The dynamic trajectories were recorded every 2 ps during the production stage for further analysis.

## Electronic supplementary material

Supplementary Dataset
