# Supplementary material for: Molecular Insights into the Interaction of RONS and Thieno[3,2-c]pyran Analogs with SIRT6/COX-2: A Molecular Dynamics Study
Source: Sci Rep. 2018 Mar 19;8:4777. doi: 10.1038/s41598-018-22972-9 (PMC5859274; doi:10.1038/s41598-018-22972-9)
Supplement: Supplementary file 1 — Supplementary Dataset [file 41598_2018_22972_MOESM1_ESM.doc]

**Molecular Insights into the Interaction of RONS and Thieno[3,2-c]pyran Analogs with SIRT6/COX-2: A Molecular Dynamics Study**

Dharmendra K. Yadav^1*#^, Surendra Kumar^1*#^, Saloni^1^, Sanjeev Misra^2^, Lalit Yadav^3^, Mahesh Teli^4^, Praveen Sharma^2^, Sandeep Chaudhary^3^, Naresh Kumar^5^, Eun Ha Choi^5^, Hyung Sik Kim^6^, Mi-hyun Kim^1#^

^1^College of Pharmacy, Gachon University, 191, Hambangmoe-ro, Yeonsu-gu, Incheon, 21936, South Korea

^2^Department of Biochemistry, All India Institute of Medical Science, Jodhpur, Rajasthan, 342005, India

^3^Department of Chemistry, Malaviya National Institute of Technology, Jawaharlal Nehru Marg, Jaipur-302017, India.

^4^Faculty of Biochemistry and Molecular Medicine Aapistie, University of Oulu, 7 A, 90220 Oulu, Finland

^5^Plasma Bioscience Research Center/ Department of Electrical and Biological Physics, Kwangwoon University, 20 Kwangwon-Ro, Nowon-Gu, Seoul 139-701, Korea

^6^School of Pharmacy, Sungkyunkwan University, Suwon 16419, Republic of Korea

^#^E-mail: [dharmendra30oct@gmail.com](mailto:dharmendra30oct@gmail.com), kmh0515@gachon.ac.kr

*contributed equally

**Corresponding Author**

Dr. Dharmendra Kumar Yadav, Ph.D

Research Professor

College of Pharmacy,

Gachon University of Medicine and Science

Room # 502, Hambakmoeiro 191, Yeonsu-gu, Incheon city, 406-799, Korea

E-mail: [dharmendra30oct@gmail.com](mailto:dharmendra30oct@gmail.com)

Office: +82-32-820-4947

**Table S1**. Compliance of designed thieno[3,2-c]pyran analogues and standard drugs to the computational parameters of pharmacokinetics (ADME).

| **Compounds** | **Aqueous solubility** | **CYP2D6 binding** | **Hepatotoxicity** | **Absoprtion level** | **Plasma Protein binding** |
| --- | --- | --- | --- | --- | --- |
| **01** | 2 (low) | False (non-inhibitor) | True (toxic) | 0 (Good) | True (Highly bounded) |
| **02** | 2 (low) | False (non-inhibitor) | True (toxic) | 0 (Good) | True (Highly bounded) |
| **02** | 3 (good) | False (non-inhibitor) | True (toxic) | 0 (Good) | True (Highly bounded) |
| **04** | 2 (low) | False (non-inhibitor) | True (toxic) | 0 (Good) | True (Highly bounded) |
| **05** | 2 (low) | False (non-inhibitor) | True (toxic) | 0 (Good) | True (Highly bounded) |
| **06** | 3 (good) | False (non-inhibitor) | True (toxic) | 0 (Good) | True (Highly bounded) |
| **07** | 2 (low) | False (non-inhibitor) | True (toxic) | 0 (Good) | True (Highly bounded) |
| **08** | 2 (low) | False (non-inhibitor) | True (toxic) | 0 (Good) | True (Highly bounded) |
| **09** | 2 (low) | False (non-inhibitor) | True (toxic) | 0 (Good) | True (Highly bounded) |
| **10** | 2 (low) | False (non-inhibitor) | True (toxic) | 0 (Good) | True (Highly bounded) |
| **11** | 1 (poor) | False (non-inhibitor) | True (toxic) | 0 (Good) | True (Highly bounded) |
| **12** | 2 (low) | False (non-inhibitor) | True (toxic) | 0 (Good) | True (Highly bounded) |
| **Celecoxib** | 2 (low) | False (non-inhibitor) | True (toxic) | 0 (Good) | True (Highly bounded) |
| **Diclofenac** | 2 (low) | True (True -inhibitor) | True (toxic) | 0 (Good) | True (Highly bounded) |
| **Flurbiprofen** | 2 (low) | False (non-inhibitor) | True (toxic) | 0 (Good) | True (Highly bounded) |
| **Indomethacin** | 2 (low) | False (non-inhibitor) | True (toxic) | 0 (Good) | True (Highly bounded) |
| **Olaparib** | 3 (good) | False (non-inhibitor) | True (toxic) | 0 (Good) | True (Highly bounded) |

**Table S2: Binding affinity of designed thieno[3,2-c] pyran analogues, standard drugs and RONS against SIRT6 (PDB ID: 3K35) enzyme.**

| **Compounds/ RONS** | **Total Score** | **Amino acid involved in active pocket in 3 Å** | **Involved group of Amino Acid** | **Length of H-bond Å** | **No. of H-Bond** |
| --- | --- | --- | --- | --- | --- |
| **1** | 5.2478 | Ala-51, Gly-52, Phe-62, Arg-63, Val-113, His-131, Leu-184, Thr-213, Ser-214, Ile-217 | Arg-63 | 1.9, 2.1 | 2 |
| **2** | 5.2196 | Gly-50, Ala-51, Phe-62, Arg-63, Trp-69, Gln-111, Asn-112, Val-113, Met-155, Gly-156, Ile-183, Asp-185, Thr-213, Ser-214 | Gln-111, Asn-112 | 2.7, 2.6 | 2 |
| **3** | 4.8502 | Ala-51, Phe-62, Gln-111, Asn-112, Val-113, Met-155, Gly-156, Ile-183, Asp-185, Trp-186, Thr-213, Ser-214 | Ala-51, Gln-111, Asn-112, | 1.8, 2.6, 2.0 | 3 |
| **4** | 5.3526 | Ala-51, Gly-52, Phe-62, Arg-63, Gln-111, Asn-112, His-131, Ser-214, Ile-217 | His-131 | 2.4 | 1 |
| **5** | 5.0923 | Ala-51, Phe-62, Arg-63, Trp-69, Gln-111, Asn-112, Val-113, Met-155, Ile-183, Ser-214, Gln-216 | Arg-63, Gln-111, | 2.1, 2.6 | 2 |
| **6** | 6.0956 | Gly-50, Ala-51, Phe-62, Arg-63, Gln-111, Ile-183, Asp-185, Trp-186, Thr-213, Ser-214, Ile-217 | Arg-63, Ala-51 | 1.8, 1.9, 2.0 | 3 |
| **7** | 6.0052 | Ala-51, Gly-52, Phe-62, Arg-63, Gln-111, Asn-112, His-131, Thr-213, Ser-214, Ile-217 | Ala-51, His-131 | 2.3, 2.6 | 2 |
| **8** | 4.8363 | Gly-50, Ala-51, Phe-62, Arg-63, Trp-69, Gln-111, Asn-112, Val-113, Met-155, Ile-183, Ser-214, Gln-216 | Arg-63, Gln-111 | 2.1, 2.4 | 2 |
| **9** | 5.5845 | Ala-51, Gly-52, Asp-61, Phe-62, Arg-63, Gln-111, Asn-112, His-131, Ser-214, Ile-217 | Ala-51, His-131 | 2.5, 2.6 | 2 |
| **10** | 6.0431 | Phe-62, Arg-63, Trp-69, Gln-111, Met-155, Lys-158, Gly-156, Leu-184, Asp-185, Trp-186, Ile-217 | Arg-63 | 1.9, 2.0 | 2 |
| **11** | 5.5806 | Ala-51, Phe-62, Arg-63, Trp-69, Gln-111, Asn-112, Val-113, Ile-183, Leu-184, Asp-185, Trp-186, Thr-213, Ser-214 | Gln-111, Asn-112 | 2.6, 2.6 | 2 |
| **12** | 5.7252 | Gly-50, Ala-51, Phe-62, Arg-63, Trp-69, Gln-111, Asn-112, Val-113, Ile-183, Leu-184, Asp-185, Trp-186, Ser-214, Gln-216, Ile-217 | Arg-63, Gln-111, Asn-112 | 2.3, 2.6, 2.0 | 3 |
| **Olaparib** | 8.3569 | Lys-13, Ala-51, Arg-63, Trp-69, Asn-112, Val-113, His-131, Ile-183, Leu-184, Asp-185, Trp-186 | Lys-13, Arg-63 | 1.7, 2.0, 2.3 | 3 |
| **H2O2** | 3.2869 | Val-113, His-131, Ile-183, Leu-184 | Leu-184 | 1.9 | 1 |
| **NO_2_** | 4.3973 | Arg-63, Gln-111, His-131, Ile-217 | Arg-63, Gln-111 | 2.3, 2.0, 2.1 | 3 |
| **N2O** | 3.4732 | Phe-62, Arg-63, Trp-69 | Arg-63, Trp-69 | 2.1, 2.5, 1.9 | 3 |
| **NO_3_** | 5.2070 | Arg-63, Gln-111, His-131, Ile-217 | Arg-63, Gln-111 | 2.3, 2.0, 2.1 | 3 |
| **N_2_O_3_** | 4.6042 | Phe-62, Arg-63, Trp-69 | Arg-63, Trp-69 | 2.3, 1.9, 1.9 | 3 |
| **N2O5** | 3.9599 | Arg-63, Trp-69, Gln-111, His-131, Ile-217 | Arg-63, Trp-69, Gln-111 | 2.0, 1.9, 2.0, 2.0 | 4 |

Note: Surﬂex-Dock scores (total scores) were expressed in - log 10 (Kd) units to represent binding affinities.

**Table S3: Binding affinity of designed thieno[3,2-c] pyran analogues, standard drugs and RONS against COX (PDB ID: 6COX) enzyme.**

| **Compounds/ RONS** | **Total Score** | **Amino acid involved in active pocket in 3 Å** | **Involved group of Amino Acid** | **Length of H-bond Å** | **No. of H-Bond** |
| --- | --- | --- | --- | --- | --- |
| **1** | 1.1043 | His-90, Thr-94, Gln-192, Val-349, Leu-352, Ser-353, Pro-514, Asp-515, Ala-516, Phe-518, Val523, Ala527, Ser-530, Leu-531 | - | - | - |
| **2** | 0.6643 | His-90, Thr-94, Gln-192, Leu-352, Ser-353, Tyr-355, Phe-381, Tyr-385, Trp-387, Ala-516, Ile-517, Phe-518, Val523, Gly-526, Ala527, Ser-530 | - | - | - |
| **3** | 1.2920 | His-90, Thr-94, Arg-120, Gln-192, Val-349, Leu-352, Ser-353, Tyr-355, Asp-515, Ala-516, Ile-517, Phe-518, Val523, Ala527, Ser-530, Leu-531 | Gln-192, Leu-352 | 1.9, 2.5 | 2 |
| **4** | 2.5087 | His-90, Val-344, Val-349, Val-523, Leu-352, Ser-353, Tyr-355, Phe-381, Leu-384, Tyr-385, Trp-387, Arg-513, Ala-516, Ile-517, Phe-518, Val523, Ser-530, Leu-534 | Ser-530 | 2.1 | 1 |
| **5** | 1.4915 | His-90, Met-113, Val-116, Leu-117, Arg-120, Gln-192, Leu-359, Leu-352, Ser-353, Tyr-355, Arg-513, Ala-516, Ile-517, Phe-518, Val523, Ala527, Leu-531 | Ala-516 | 1.9 | 1 |
| **6** | 6.8541 | His-90, , Val-349, Leu-352, Ser-353, Tyr-355, Phe-381, Tyr-385, Trp-387, Ala-516, Ile-517, Phe-518, Val-523, Ser-530, Leu-534 | Ser-530 | 1.7 | 1 |
| **7** | 3.2769 | His-90, Tyr-348, Val-349, Val-523, Leu-352, Ser-353, Tyr-355, Phe-381, Tyr-385, Trp-387, Ala-516, Ile-517, Phe-518, Gly-526, Ser-530, Leu-534 | - | - | - |
| **8** | 4.5911 | His-90, Tyr-348, Val-349, Val-523, Leu-352, Ser-353, Tyr-355, Phe-381, Tyr-385, Trp-387, Ala-516, Ile-517, Phe-518, Gly-526, Ser-530, Leu-534 | Ser-353 | 2.6 | 1 |
| **9** | 5.0961 | His-90, Val, 116, Met113, Val-349, Leu-352, Ser-353, Tyr-355, Leu-359, Arg-513, Ala-516, Ile-517, Phe-518, Val-523, Ala-527, Leu-531 | - | - | - |
| **10** | 6.8841 | His-90, Val-344, Val-349, Leu-352, Ser-353, Tyr-355, Phe-381, Leu-384, Tyr-385, Trp-387, Arg-513, Ala-516, Ile-517, Phe-518, Met-522, Val-523, Gly-526, Ser-530, Leu-534 | Ser-530 | 1.9 | 1 |
| **11** | 4.0521 | His-90, Thr-94, Gln-192, His-351, Ser-353, Gly-354, Arg-513, Pro-514, Ala-516, Phe-518, Val523 | - | - | - |
| **12** | 6.7892 | His-90, Gln-192, Tyr-348, Val-349, Leu-352, Ser-353, Tyr-355,  Phe-381, Leu-384, Tyr-385, Trp-387, Arg-513, Ala-516, Ile-517, Phe-518, Val523, Gly-526, Ala527, Ser-530, Leu-534 | Ser-530, Gly-526 | 2.2, 2.5 | 2 |
| **Flurbiprofen** | 6.1641 | His-90, Gln-192, Val-349, Leu-352, Ser-353, Tyr-355, Arg-513,  Ala-516, Ile-517, Phe-518, Val-523, Ala-527, Leu-531 | Phe-518 | 1.8 | 1 |
| **Celecoxib** | 6.3151 | Met-113, Val-116, Trp-348, Val-349, Leu-352, Ser-353, Tyr-355, Leu-359, Tyr-385, Val-523, Gly-526, Ala-527, Ser-530, Leu-531 | - | - | - |
| **H2O2** | 3.1340 | His-90, Ser-353, Tyr-355 | His-90, Ser-353 | 2.0, 2.0 | 2 |
| **NO_2_** | 2.5823 | Val-349, Ser-530 | Ser-530 | 1.9, 2.6 | 2 |
| **N2O** | 2.3965 | Val-349, Ser-530 | Ser-530 | 1.9 | 1 |
| **NO3** | 3.2150 | Val-349, Leu-531, Ser-530 | Ser-530 | 2.6, 2.6, 1.9 | 3 |
| **N2O3** | 3.0906 | Arg-120, Ala-527, Val-523, Ser-353, Tyr-355 | Arg-120 | 1.9, 2.3 | 2 |
| **N2O5** | 1.6145 | His-90, Arg-513, Ala-516, Ile-517, Phe-518, Val-523 | Arg-513, Phe-518 | 2.1, 1.9 | 2 |

Note: Surﬂex-Dock scores (total scores) were expressed in - log 10 (Kd) units to represent binding affinities.
